# Influence of the hiking tourscape on hikers’ psychological restoration: the distinct mediating roles of place dependence and place identity

**DOI:** 10.3389/fpsyg.2026.1889577

**Published:** 2026-08-19

**Authors:** Yan-Ting Wang, Guo-Liang Lin, Yi Chen Chiu

**Affiliations:** 1 School of Physical Education, Quanzhou Normal University, Quanzhou, China; 2 Faculty of Education, City University of Malaysia, Petaling Jaya, Malaysia; 3 Department of Leisure and Recreation Management, Taipei City University of Science and Technology, Taipei, Taiwan

**Keywords:** hiking tourscape, hospitality culture, place dependence, place identity, psychological restoration

## Abstract

**Background:**

Hiking restoration may be shaped not only by exposure to nature but also by the atmospheric, interpersonal, and activity-related features encountered along a route. This study examined how four dimensions of hiking tourscape, namely natural dimension, atmosphere dimension, hospitality culture, and experiential activities, were associated with psychological restoration. It further investigated whether place dependence and place identity provided distinct mediating pathways.

**Methods:**

A cross-sectional survey was conducted with 452 hikers at two hiking destinations in Zunyi, China. The measurement structure was examined using principal component analysis and confirmatory factor analysis. Structural equation modelling was used to test the hypothesised direct relationships, and bootstrap analysis with 5,000 resamples was conducted to evaluate the specific indirect effects through place dependence and place identity.

**Results:**

Natural dimension, atmosphere dimension, hospitality culture, and experiential activities were all positively associated with psychological restoration, with hospitality culture showing the strongest direct association. Natural dimension, atmosphere dimension, and hospitality culture were positively associated with place dependence, whereas experiential activities were not. Place dependence showed a marginal but nonsignificant direct association with psychological restoration, while place identity was not significantly associated with psychological restoration. The specific indirect effects through place dependence were significant for natural dimension, atmosphere dimension, and hospitality culture, but not for experiential activities. In contrast, none of the four hiking tourscape dimensions was significantly associated with place identity, and no indirect effects through place identity were identified.

**Conclusion:**

Restorative hiking experiences are associated with a multidimensional combination of natural, atmospheric, interpersonal, and activity-related features. The findings also show that place dependence and place identity perform different psychological functions. Functional suitability provided a selective pathway linking several tourscape dimensions with restoration, whereas symbolic identification did not provide an additional explanatory pathway in the present sample. These findings support treating place attachment as a multidimensional rather than uniform mechanism. Given the cross-sectional design, convenience sampling, and predominantly young and university-educated sample, causal and broader generalisations should be made cautiously.

## 1 Introduction

Hiking tourism has become an increasingly prominent form of leisure and nature based recreation worldwide (UNWTO, 2019). It involves walking through natural landscapes, historical sites, rural paths, and mountain routes for exercise, recreation, cultural exploration, or psychological relaxation (Gómez-Martín, 2019), and is commonly developed through walking, trekking, and mountaineering products (Muhar et al., 2007). By bringing participants into sustained contact with natural and cultural environments, hiking has been associated with physical health, psychological well being, and recovery from everyday demands (Abraham et al., 2010; Geiger et al., 2023; Gross and Sand, 2019). Research in this area, however, has largely emphasised physiological outcomes and behavioural characteristics. Physiologically oriented studies have examined cardiovascular responses, physical fitness, muscular endurance, and other health related indicators (Gatterer et al., 2015; Kang, 2014; Mitten et al., 2018), while tourism and recreation studies have focused on participation motives, trail conditions, route preferences, and observable hiking behaviour (Bichler and Peters, 2021; Gómez-Martín, 2019; Molokáč et al., 2022; Taczanowska et al., 2014).

Although these perspectives provide important evidence regarding the benefits of hiking and the ways in which people use outdoor environments, they offer less insight into how hikers experience the environment as a combined configuration of sensory, atmospheric, interpersonal, and activity related features. Physiological studies typically operationalise hiking outcomes through measurable bodily responses or performance indicators, treating visual, auditory, tactile, atmospheric, and social cues largely as contextual conditions rather than explanatory variables. Behavioural studies similarly prioritise observable choices, movements, motivations, and route use patterns rather than the integration of environmental, affective, interpersonal, and participatory cues during the hiking experience. As a result, existing research provides limited understanding of how the broader experiential environment of hiking is associated with psychological restoration.

This limitation is important because psychological restoration may depend on more than exposure to nature alone. Visual, auditory, tactile, affective, and social qualities can all influence psychological well being and recovery (Malekinezhad et al., 2020; Song et al., 2025). Recent studies have begun to examine sensory dimensions and broader experiencescape qualities in green space and active tourism settings, but much of this research has concentrated on relatively static environments, general experiential value, or perceived restorativeness as an overall environmental evaluation (Malekinezhad et al., 2020; Song et al., 2025). Hiking is different because it unfolds progressively through movement. Hikers encounter changing scenery, terrain, atmosphere, human support, and activity opportunities along a route, making the experience mobile, embodied, and processual rather than confined to a single location.

The concept of hiking tourscape provides a context sensitive framework for examining these features together. Drawing on servicescape and tourism experience research, hiking tourscape refers to the environmental, atmospheric, interpersonal, and activity related stimuli perceived through direct engagement with a hiking destination. In the present study, it comprises four dimensions: natural dimension, atmosphere dimension, hospitality culture, and experiential activities.

The theoretical value of hiking tourscape lies in its ability to integrate several features that are only partially captured by related concepts. Servicescape emphasises the influence of designed service environments on consumers’ emotional and behavioural responses, but it is commonly applied to relatively bounded and stable service settings (Bitner, 1992; Rosenbaum, 2005). Destination image focuses on visitors’ broader cognitive and affective representations of a destination, but it does not necessarily capture the situated and sequential experience of moving through a trail (Baloglu and McCleary, 1999; Echtner and Ritchie, 1993). Restorative environment research explains why certain settings support attentional recovery and stress reduction, but it often treats restoration as a property of the environment or as a general perceived quality rather than examining the specific experiential components that produce restoration (Kaplan and Kaplan, 1989; Kaplan, 1995; Ulrich et al., 1991). Nature based tourism experience highlights contact with natural settings, but it may give less attention to the combined role of atmosphere, interpersonal support, and activity opportunities (Gómez-Martín, 2019; Mehmetoglu, 2007; Pomfret and Bramwell, 2016). By contrast, hiking tourscape captures the route based configuration of natural, atmospheric, interpersonal, and activity related cues that hikers encounter through bodily movement and direct participation. It therefore provides a more context sensitive framework for explaining how different elements of hiking environments jointly contribute to psychological restoration and place related responses.

Attention Restoration Theory, Stress Recovery Theory, and place attachment theory provide complementary explanations for the relationships between hiking tourscape and psychological restoration. Attention Restoration Theory proposes that environments characterised by fascination, compatibility, extent, and a sense of being away can support recovery from directed attention fatigue (Kaplan and Kaplan, 1989; Kaplan, 1995), whereas Stress Recovery Theory emphasises stress reduction and positive affective responses during exposure to supportive environmental conditions (Ulrich et al., 1991). In hiking settings, natural scenery, a pleasant atmosphere, supportive interpersonal encounters, and absorbing activities may therefore contribute directly to restoration through cognitive, emotional, social, and participatory processes. Place attachment theory adds a relational explanation by showing how environmental experiences may become functionally valuable or personally meaningful. Place dependence concerns the functional suitability and comparative preference of a setting, whereas place identity concerns the symbolic, emotional, and self related meanings associated with a place (Williams et al., 1992; Williams and Vaske, 2003). In this integrated framework, hiking tourscape dimensions may influence psychological restoration directly through sensory, affective, interpersonal, and activity related experiences, and they may also operate indirectly by strengthening place dependence or place identity.

Existing studies have connected environmental perception, place attachment, and restoration in historical districts, rural tourism settings, and hiking destinations (Li et al., 2023; Tang et al., 2024; Yoon et al., 2023). However, they have generally operationalised the environment through broad measures such as landscape perception, naturalness, or perceived restorativeness and have differed in the ordering of environmental perception, attachment, and restoration. They therefore provide less evidence regarding whether natural, atmospheric, interpersonal, and activity related features of hiking environments are associated with psychological restoration directly, or whether they operate through different dimensions of place attachment. This issue is particularly relevant in China, where hiking tourism has expanded alongside the National Trail System and fitness tourism initiatives associated with the Healthy China 2030 strategy (Zhong et al., 2023), but empirical research on the psychological and emotional dimensions of hiking tourism remains limited.

The unresolved issue is thus not simply whether environmental perception, person place relationships, and restoration are related, but how distinct components of a route based hiking experience are associated with immediate psychological restoration and with different forms of attachment to place. To address this issue, the present study examines the direct associations of four hiking tourscape dimensions, including natural dimension, atmosphere dimension, hospitality culture, and experiential activities, with psychological restoration. It also evaluates their relationships with place dependence and place identity and tests whether these two constructs provide distinct mediating pathways. By distinguishing functional dependence from symbolic identification, the study offers a more precise account of how hiking environments may be related to restorative experience and clarifies whether place attachment operates as a uniform mechanism or as a set of differentiated psychological processes.

## 2 Literature review

### 2.1 Hiking tourscape

The concept of servicescape originated in research examining how designed environments shape customers’ emotions and behavioural responses. Kotler (1973) described the consumption environment as a setting intentionally arranged to evoke particular feelings and behaviours, while Bitner (1992) conceptualised servicescape in terms of ambient conditions, spatial layout, and symbolic artefacts. Rosenbaum (2005) later extended this perspective by emphasising the cultural and symbolic meanings embedded in service settings. As the literature moved beyond indoor and commercially bounded environments, increasing attention was given to the broader physical, atmospheric, and social conditions through which visitors experience destinations (Choo and Petrick, 2014; Torres-Moraga et al., 2024).

This development contributed to experiential landscape and tourscape perspectives. O’Dell (2005) proposed that tourism experiences emerge through interactions among physical surroundings, products, social settings, and other people, while Mossberg (2007) similarly highlighted the experiential environment surrounding tourism consumption. Tourscape therefore refers to the configuration of environmental and social stimuli perceived through tourists’ engagement with a destination (Zhang and Xu, 2019). Its dimensions may be physical, natural, atmospheric, interpersonal, or symbolic and have been examined in ski, nature based, mountain, wellness, and night tourism settings (Fossgard and Fredman, 2019; McLeay et al., 2019; Vespestad and Hansen, 2020; Chen et al., 2023; Ruan et al., 2023).

Hiking requires a context sensitive application of tourscape because the experience unfolds through movement. Hikers encounter scenery, terrain, atmosphere, other people, and activity opportunities progressively through walking, bodily effort, pauses, and changes along the route (Dorwart et al., 2009; Peterson et al., 2018; Skov-Petersen et al., 2021; Taczanowska et al., 2014). Hiking tourscape therefore extends servicescape beyond a relatively bounded service environment. It also differs from destination image, which primarily concerns broader cognitive and affective representations of a destination, and from restorative environment research, which generally evaluates whether a setting supports attentional recovery or stress reduction. In the present study, psychological restoration is treated as an outcome to be explained through hikers’ perceptions of specific environmental and experiential features.

Existing studies provide only partial coverage of this configuration. Li et al. (2023) examined overall landscape perception in historical districts, Tang et al. (2024) focused on naturalness and the spatial and formal qualities of rural environments, and Yoon et al. (2023) positioned perceived restorativeness as an antecedent of place attachment among hikers. These studies establish important relationships among environmental perception, person place bonds, and restoration, but provide less insight into how atmospheric conditions, supportive interpersonal encounters, and activity opportunities operate alongside natural features. Research on hiking and outdoor recreation nevertheless suggests that trail experiences are shaped by route conditions, situational qualities, interpretive support, signage, management, nature observation, and opportunities for active participation (Greer et al., 2017; Kuldna et al., 2020; Pomfret et al., 2023; Rice et al., 2023; Yang, 2024).

Building on this literature, the present study conceptualises hiking tourscape through four dimensions. The natural dimension concerns the scenic and ecological qualities encountered along the route, including vegetation, terrain, and the wider landscape. The atmosphere dimension captures the affective and situational character of the setting, including ambience, emotional tone, comfort, and perceived pace. Hospitality culture refers to supportive interpersonal and service related features, such as friendliness, welcoming attitudes, interpretive assistance, and trail guidance (Tasci and Pizam, 2020). Experiential activities represent opportunities for participatory engagement beyond walking alone, including photography, nature observation, outdoor activities, and other place based experiences.

Attention Restoration Theory and Stress Recovery Theory provide complementary explanations for why these dimensions may be associated with psychological restoration. ART proposes that environments characterised by soft fascination, compatibility, extent, and a sense of being away can restore directed attention (Kaplan and Kaplan, 1989; Kaplan, 1995). SRT suggests that supportive environmental conditions may reduce stress and promote positive affective responses (Ulrich et al., 1991). Within a hiking context, the four dimensions of tourscape may therefore contribute to restoration through different but related experiential processes.

Natural environments have consistently been associated with stress reduction, attentional recovery, subjective well being, and psychological detachment from everyday demands (MacKerron and Mourato, 2013; Ulrich et al., 1991). Mountains, forests, vegetation, terrain, and changing scenery may provide sustained opportunities for fascination, sensory engagement, and emotional relief during hiking (Huang et al., 2020; Kaplan, 1995).

*H1a*: The natural dimension of the hiking tourscape is positively associated with hikers’ psychological restoration.

Atmospheric qualities such as peacefulness, comfort, openness, safety, and a leisurely pace may shape the emotional tone of the hiking experience. Positive environmental atmospheres have been associated with emotional regulation, perceived restorativeness, and psychological comfort (Korpela and Hartig, 1996; Korpela et al., 2017; Yu et al., 2021). A calm and pleasant atmosphere may therefore help hikers disengage from daily pressures and facilitate recovery.

*H1b*: The atmosphere dimension of the hiking tourscape is positively associated with hikers’ psychological restoration.

Hospitality culture represents the interpersonal and service related qualities of the destination. Friendly, attentive, and supportive interactions may reduce uncertainty, strengthen perceived safety, and increase emotional comfort during tourism experiences. Previous studies have linked hospitable service environments with positive affect, emotional well being, and more favourable destination experiences (Abdulaziz et al., 2023; Gao and Tong, 2010).

*H1c*: Hospitality culture is positively associated with hikers’ psychological restoration.

Experiential activities may support restoration by increasing absorption, novelty, fascination, and temporary detachment from routine concerns. Activities such as scenic viewing, photography, nature observation, and other forms of place based engagement may redirect attention away from everyday stressors and encourage active involvement with the destination. Participatory and sensory rich tourism experiences have been associated with positive affect and cognitive recovery (Cui et al., 2025; Kaplan, 1995; Song et al., 2025).

*H1d:* Experiential activities are positively associated with hikers’ psychological restoration.

Taken together, the literature indicates that environmental perception, place attachment, and restoration are related, but it does not yet provide a sufficiently differentiated explanation of these relationships in route-based hiking settings. Existing studies have commonly relied on broad environmental evaluations, have given limited attention to atmospheric, interpersonal, and activity-related features, and have not consistently distinguished the functional and symbolic dimensions of place attachment. The present study addresses these limitations by examining four hiking tourscape dimensions and by comparing place dependence and place identity as distinct direct and mediating pathways to psychological restoration.

### 2.2 Place dependence and place identity

Place attachment has roots in geography, psychology, sociology, tourism, and leisure research and is widely used to explain how individuals form meaningful connections with environments. Early accounts described it as an emotional bond between individuals and places (Shumaker, 1983), while later research emphasised that person place relationships may involve functional, affective, cognitive, behavioural, symbolic, and social processes (Low and Altman, 1992; Williams et al., 1992). Scannell and Gifford (2010) integrated these perspectives within a tripartite framework comprising person, psychological process, and place.

Place attachment is generally regarded as multidimensional. Williams et al. (1992) and Williams and Vaske (2003) distinguished place dependence from place identity. Place dependence concerns the functional suitability and relative irreplaceability of a setting for supporting desired activities. It develops when a location is perceived as better able than alternative places to satisfy recreational, leisure, or restorative needs. Place identity, in contrast, concerns the symbolic, emotional, and self related meanings through which a setting becomes incorporated into an individual’s self concept.

Although both dimensions belong to the broader domain of place attachment, they represent different psychological processes. Place dependence is primarily based on functional compatibility, activity support, and comparative preference. Place identity involves symbolic meaning, memory, belonging, and self definition. Environmental quality, activity opportunities, accessibility, satisfaction, and suitable resources may strengthen place dependence, whereas memorable experiences, repeated visitation, emotional involvement, and personal memories may contribute more strongly to place identity (Hammitt et al., 2004; Kyle et al., 2004a,b; Scannell and Gifford, 2010; Sthapit et al., 2023).

These two dimensions may also contribute differently to psychological restoration. A functionally suitable place may provide reliable opportunities for exercise, relaxation, escape, and recovery. Hikers who perceive a destination as especially compatible with their needs may experience greater familiarity, confidence, and comfort when using that setting. Recreation research has shown that activity involvement and satisfaction with place based resources are closely related to place dependence (Hammitt et al., 2004; Kyle et al., 2004b), while ART identifies compatibility between environmental conditions and individual purposes as an important condition for restoration (Kaplan, 1995).

*H2a*: Place dependence is positively associated with hikers’ psychological restoration.

Place identity may contribute to restoration through symbolic meaning, emotional security, belonging, and continuity of self. Personally meaningful places can evoke memories and feelings of familiarity that increase their emotional and restorative significance. Studies of favourite and restorative places suggest that place based memories, identification, and personal significance may enhance perceived restorativeness and emotional recovery (Ratcliffe and Korpela, 2016; Subiza-Pérez et al., 2021).

*H2b*: Place identity is positively associated with hikers’ psychological restoration.

### 2.3 Hiking tourscape and place dependence

The dimensions of hiking tourscape may shape place dependence by influencing whether a destination is perceived as suitable, useful, and preferable for achieving recreational goals. Natural qualities are especially relevant because scenery, vegetation, terrain, ecological richness, and environmental tranquillity determine whether a setting can effectively support hiking, exercise, relaxation, and nature engagement. Recreation research has shown that environmental resources and activity opportunities can strengthen functional attachment when a setting satisfies users’ needs better than alternative locations (Hammitt et al., 2004; Kyle et al., 2004b,c).

*H3a*: The natural dimension of the hiking tourscape is positively associated with place dependence.

Atmospheric qualities may also shape functional evaluations of a destination. Comfort, calmness, perceived safety, openness, and a leisurely pace can influence whether visitors regard a place as suitable for relaxation and escape. Positive environmental and affective evaluations have been associated with destination preference, satisfaction, and attachment in tourism settings (Kim and Ritchie, 2014; Kucukergin et al., 2020; Zhou et al., 2024). A hiking destination that consistently supports desired emotional and recreational experiences may therefore become comparatively preferable.

*H3b*: The atmosphere dimension of the hiking tourscape is positively associated with place dependence.

Hospitality culture may strengthen place dependence by improving the practical usability and perceived suitability of a destination. Friendly staff, clear guidance, interpretive assistance, and responsive support may help visitors navigate the setting, reduce uncertainty, and participate more comfortably. Supportive interpersonal and service experiences have been associated with destination evaluation, satisfaction, and attachment (Ramkissoon et al., 2013; Yuksel et al., 2010; Zou et al., 2022).

*H3c*: Hospitality culture is positively associated with place dependence.

Experiential activities may increase the functional value of a hiking destination by expanding the range of goals that visitors can achieve there. Opportunities for scenic viewing, photography, nature observation, outdoor cooking, and other activities may increase involvement and make the destination more useful for recreation and leisure. Prior research has linked activity involvement and meaningful participation with stronger place based bonds and functional attachment (Gross and Brown, 2008; Kyle et al., 2004a; Sthapit, 2017).

*H3d*: Experiential activities are positively associated with place dependence.

### 2.4 Hiking tourscape and place identity

The dimensions of hiking tourscape may also contribute to place identity when environmental and experiential features acquire personal and symbolic meaning. Natural landscapes may become associated with memories, emotional experiences, and self related meanings through repeated, memorable, or personally significant encounters. Distinctive scenery, ecological features, and embodied engagement with nature have been linked to attachment and identification with place (Raymond et al., 2010; Scannell and Gifford, 2010; Sthapit et al., 2023).

*H4a*: The natural dimension of the hiking tourscape is positively associated with place identity.

Atmospheric qualities may influence how visitors remember and interpret a destination. A calm, joyful, distinctive, or welcoming atmosphere may generate emotionally meaningful experiences and contribute to the perception that a place is personally special. Affective and memorable tourism experiences have been associated with emotional affinity and identification with destinations (Kim and Ritchie, 2014; Kucukergin et al., 2020; Zhou et al., 2024).

*H4b*: The atmosphere dimension of the hiking tourscape is positively associated with place identity.

Hospitality culture may contribute to place identity through feelings of recognition, belonging, and social acceptance. Positive interactions with staff, guides, and service personnel may become part of visitors’ memories and symbolic understanding of a destination. Hospitable encounters, social bonding, and supportive interactions have been shown to strengthen emotional attachment and identification with tourism places (Ramkissoon et al., 2013; Yuksel et al., 2010; Zou et al., 2022).

*H4c*: Hospitality culture is positively associated with place identity.

Experiential activities may foster place identity by enabling self expression, emotional involvement, and the creation of personally meaningful memories. Participation in photography, nature observation, scenic appreciation, and locally distinctive activities may deepen engagement with a destination and contribute to its incorporation into visitors’ self related meanings. Previous studies have connected active participation, memorable tourism experiences, and experiential involvement with stronger place attachment and identification (Gross and Brown, 2008; Sthapit, 2017; Zhou et al., 2021).

*H4d*: Experiential activities are positively associated with place identity.

### 2.5 Mediating roles of place dependence and place identity

Psychological restoration in hiking tourism may arise from both immediate restorative experiences and the person and place relationships that develop through those experiences. Attention Restoration Theory and Stress Recovery Theory explain relatively immediate restorative processes. ART suggests that environments can restore directed attention when they provide fascination, compatibility, extent, and a sense of being away (Kaplan and Kaplan, 1989; Kaplan, 1995). SRT emphasises that supportive environmental conditions can reduce stress and promote positive affective responses (Ulrich et al., 1991). Within a hiking context, natural scenery may generate fascination and psychological distance from daily concerns; atmospheric comfort may support emotional regulation and relaxation; hospitable interpersonal encounters may provide reassurance, safety, and psychological security; and experiential activities may create novelty, absorption, and temporary detachment from routine pressures. These mechanisms suggest that hiking tourscape dimensions may directly contribute to psychological restoration through sensory, affective, interpersonal, and participatory experiences.

Place attachment theory adds a relational explanation by showing how environmental and experiential qualities may become functionally valuable or personally meaningful. This relational pathway is important because restorative benefits may not only occur during immediate exposure to environmental cues, but may also be strengthened when a destination is perceived as suitable, preferable, meaningful, or emotionally significant. Place attachment has commonly been conceptualised as a multidimensional construct that includes place dependence and place identity (Williams et al., 1992; Williams and Vaske, 2003). These two dimensions represent different psychological processes and may therefore provide different mediating pathways between hiking tourscape and psychological restoration.

Place dependence represents a functional pathway. It refers to the perceived suitability and comparative advantage of a setting for supporting desired activities and goals (Williams et al., 1992; Williams and Vaske, 2003). In hiking tourism, a destination may become functionally important when its natural qualities, atmosphere, services, and activity opportunities make it suitable for leisure, exercise, relaxation, and recovery. Environmental resources and activity satisfaction have been shown to strengthen functional dependence on recreational settings (Hammitt et al., 2004; Kyle et al., 2004b), while the compatibility between environmental conditions and individual purposes is also central to restorative experience in ART (Kaplan, 1995). Therefore, hiking tourscape may first strengthen visitors’ perceptions that a destination is functionally compatible with their leisure and recovery goals, and this sense of functional compatibility may then contribute to psychological restoration.

Natural qualities may strengthen place dependence by making a destination particularly suitable for exercise, nature engagement, and relaxation. Atmosphere may increase the perceived suitability of a destination for comfort and escape. Hospitality culture may improve usability, reduce uncertainty, and enhance visitors’ confidence in using the destination. Experiential activities may increase the range of recreational goals that can be achieved at the site. To the extent that these features strengthen functional preference and perceived suitability, they may contribute indirectly to psychological restoration through place dependence.

*H5a*: Place dependence mediates the association between the natural dimension of the hiking tourscape and psychological restoration.

*H5b*: Place dependence mediates the association between the atmosphere dimension of the hiking tourscape and psychological restoration.

*H5c*: Place dependence mediates the association between hospitality culture and psychological restoration.

*H5d*: Place dependence mediates the association between experiential activities and psychological restoration.

Place identity represents a symbolic and emotional pathway. It refers to the meanings, memories, emotional bonds, and self related associations through which a place becomes part of an individual’s self concept (Proshansky et al., 1983; Williams et al., 1992; Williams and Vaske, 2003). Compared with place dependence, place identity is less concerned with functional suitability and more concerned with personal meaning, belonging, emotional attachment, and continuity of self. Studies of favourite and personally meaningful places suggest that place based memories, identification, and personal significance may enhance perceived restorativeness and emotional recovery (Ratcliffe and Korpela, 2016; Scannell and Gifford, 2010; Subiza-Pérez et al., 2021).

In hiking tourism, natural landscapes may support place identity when they become associated with symbolic and personal meanings. Atmosphere may contribute to place identity by creating memorable emotional experiences. Hospitality culture may foster feelings of recognition, belonging, and social connection. Experiential activities may create opportunities for self expression, involvement, and personally significant memories. If these environmental and experiential features become incorporated into visitors’ symbolic understanding of the destination, place identity may provide an additional pathway through which hiking tourscape contributes to psychological restoration.

*H6a*: Place identity mediates the association between the natural dimension of the hiking tourscape and psychological restoration.

*H6b*: Place identity mediates the association between the atmosphere dimension of the hiking tourscape and psychological restoration.

*H6c*: Place identity mediates the association between hospitality culture and psychological restoration.

*H6d*: Place identity mediates the association between experiential activities and psychological restoration.

**Figure 1:**
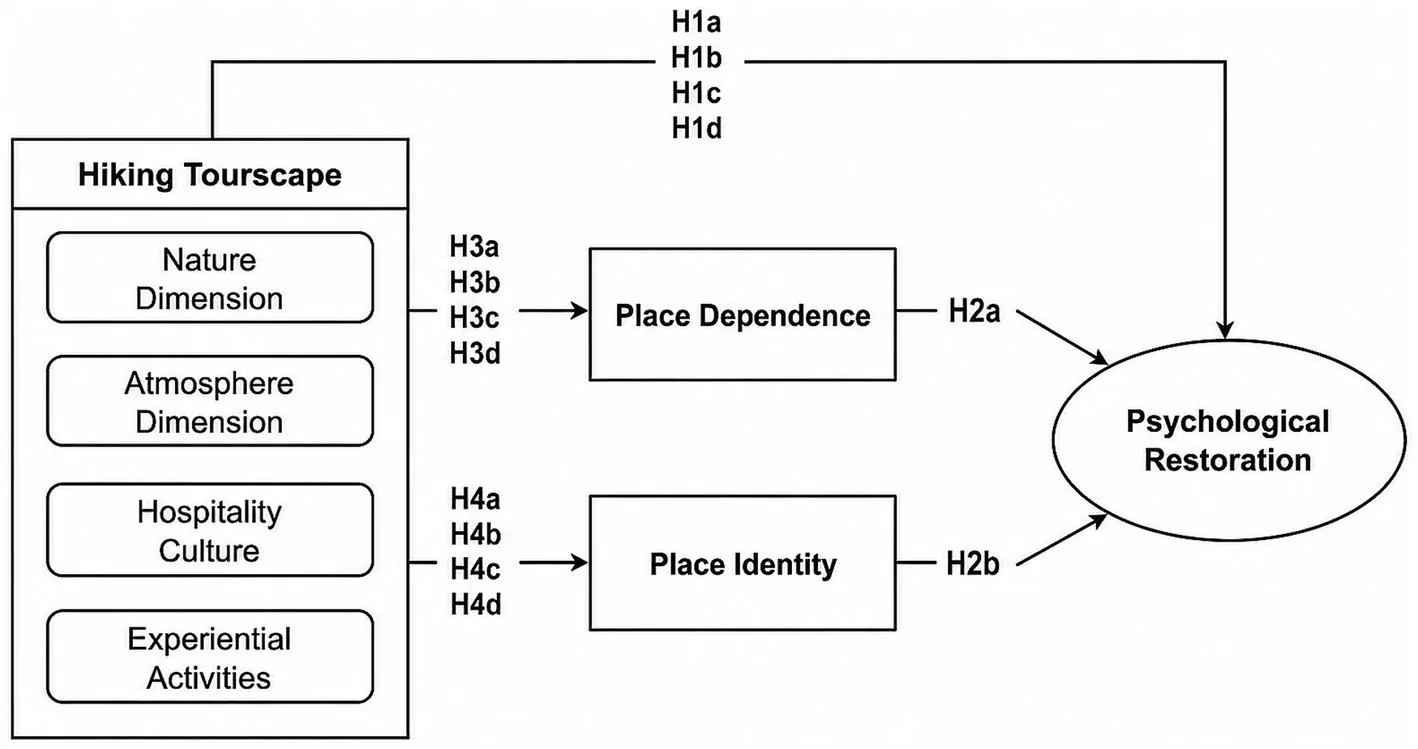
Conceptual model.

The conceptual model of the study is presented in Figure 1.

## 3 Materials and methods

This study adopted a cross-sectional quantitative design to examine the relationships among the dimensions of hiking tourscape, place dependence, place identity, and psychological restoration. A quantitative approach was appropriate because it enabled data to be collected from a relatively large sample and allowed the proposed relationships to be examined systematically through statistical analysis (Polit and Beck, 2010). Survey data were collected from hikers at two destinations in China and analysed using exploratory factor analysis, confirmatory factor analysis, and structural equation modelling. Although this design provided structured empirical evidence for testing the proposed model, the findings should be interpreted within the limitations of cross-sectional and convenience-sampling data (Stockemer et al., 2019).

### 3.1 Research measures

The questionnaire comprised two sections. The first section collected demographic information, including gender, age, monthly income, and educational level. The second section measured seven constructs derived from the conceptual framework: natural dimension, atmosphere dimension, hospitality culture, experiential activities, place dependence, place identity, and psychological restoration.

All constructs were assessed using multi-item scales adapted from previously validated instruments and rated on a five-point Likert scale ranging from 1 = strongly disagree to 5 = strongly agree. The wording of selected items was adjusted to suit the context of hiking tourism in China while retaining the intended conceptual meaning of the original measures. Place dependence and place identity were treated as two empirically distinct dimensions within the broader conceptual domain of place attachment. Table 1 summarises the number of items and primary measurement sources for each construct.

**Table 1:** Measurement sources of the study constructs.

Construct	Number of items	Primary measurement source
Natural dimension	5	Rosenbaum (2005) and Rosenbaum et al. (2016)
Atmosphere dimension	4	Radic et al. (2021)
Hospitality culture	3	Tasci and Pizam (2020)
Experiential activities	5	Ruan et al. (2023)
Place dependence	3	Williams and Vaske (2003)
Place identity	5	Williams and Vaske (2003)
Psychological restoration	7	Staats et al. (2003) and Wang et al. (2021)

The hiking tourscape items captured respondents’ perceptions of environmental, atmospheric, interpersonal, and activity-related features encountered or observed at the destination. They were not intended to indicate that every respondent had personally participated in every available activity or interacted extensively with service personnel. This approach is consistent with the conceptualisation of tourscape as the perceived configuration of destination cues rather than as a record of individual participation in each activity. Accordingly, the experiential-activities measure reflected respondents’ perceptions of the range and availability of activity opportunities within the hiking setting, whereas the hospitality-culture measure reflected the observable availability and perceived quality of human support, including visitor assistance, interpretation, guidance, and other hiking-related services. The reliability and validity of the resulting seven-factor measurement structure were subsequently assessed through exploratory and confirmatory factor analyses.

#### 3.1.1 Natural dimension

The natural dimension was measured using five items adapted from Rosenbaum (2005) and Rosenbaum et al. (2016). The items assessed respondents’ perceptions of scenic attractiveness, ecological richness, the integration of natural and cultural landscape features, a sense of escape from everyday life, and the extent to which the scenery met their expectations. These indicators capture both affective and cognitive evaluations of the natural environment and are consistent with the restorative role of nature proposed in Stress Recovery Theory and Attention Restoration Theory (Ulrich et al., 1991; MacKerron and Mourato, 2013). The expectation-related item was interpreted as an evaluation of perceived natural-environment quality rather than as a measure of overall destination satisfaction.

#### 3.1.2 Atmosphere dimension

The atmosphere dimension was measured using four items adapted from Radic et al. (2021). The items assessed respondents’ perceptions of the interactive, joyful, and leisurely qualities of the hiking setting, together with the pace of life experienced at the destination. Collectively, these indicators capture the affective and situational tone of the trail environment and the extent to which it was perceived as pleasant and relaxing.

#### 3.1.3 Hospitality culture

Hospitality culture was measured using three items adapted from Tasci and Pizam (2020). The items assessed respondents’ perceptions of the friendliness, enthusiasm, and attentiveness of personnel involved in visitor assistance, interpretation, guidance, and other hiking-related services. Such personnel were present at both study sites and formed part of the destinations’ observable human-support environment. Because the degree of direct interaction varied among visitors, particularly for those hiking independently, the construct was interpreted as reflecting the perceived availability and quality of interpersonal support rather than the extent of personal contact with service staff.

#### 3.1.4 Experiential activities

Experiential activities were measured using five items adapted from Ruan et al. (2023). The items covered opportunities for nature appreciation, camping or outdoor cooking, photography, river tracing, and tasting local agricultural products. Because the wording focused on what the destination offered or made available, the construct was interpreted as respondents’ perceptions of experiential richness and activity opportunities within the hiking setting. It did not require each respondent to have personally participated in every listed activity, as the relevance and use of these opportunities could vary across routes, visitor preferences, and visit purposes.

#### 3.1.5 Place dependence

Place dependence was measured using three items adapted from Williams and Vaske (2003). The items assessed the functional suitability and comparative preference of the hiking destination, including whether the destination was considered suitable for leisure and relaxation, preferred over alternative places, and better able to meet respondents’ recreational needs through its facilities. These items reflect the extent to which the destination was perceived as functionally valuable and relatively difficult to substitute for desired hiking and leisure experiences. The wording was contextually adapted to hiking tourism in China and administered in Chinese.

#### 3.1.6 Place identity

Place identity was measured using five items adapted from Williams and Vaske (2003). The items assessed place specific satisfaction, identification with the destination, its special personal meaning, emotional attachment, and attraction to its distinctive lifestyle and customs. Together, these indicators reflect the symbolic, emotional, and self related meanings associated with the hiking destination.

One item concerned the sense of satisfaction provided by the destination relative to other places, while the remaining items more directly addressed identification, personal significance, emotional attachment, and attraction to local characteristics. The exploratory factor analysis indicated that these five items formed a distinct place identity factor. They were therefore retained as indicators of place identity in the subsequent confirmatory factor and structural analyses. The items were contextually adapted to hiking tourism in China and administered in Chinese.

#### 3.1.7 Psychological restoration

Psychological restoration was measured using seven items adapted from Staats et al. (2003) and Wang et al. (2021). The items assessed respondents’ perceived recovery following the hiking experience, including relief from stress, physical and mental balance, inner peace, comfort, temporary release from worries, renewed energy, and vitality. Collectively, these indicators reflect the affective and attentional recovery processes described in Stress Recovery Theory and Attention Restoration Theory.

### 3.2 Data collection and research sample

This study used on-site convenience sampling at two major hiking destinations in Zunyi, China: Loushanguan and Hongjun Mountain. A total of 452 valid responses were obtained, all completed on-site via QR code scanning using participants’ mobile devices.

Data collection took place between October 2024 and January 2025. During this period, survey administrators received targeted training and were selected based on their professional background relevant to the research domain, ensuring they could effectively assist respondents with any questions. Among those who agreed to participate, 470 questionnaires were distributed and returned. After eliminating 18 invalid responses due to incomplete answers, 452 valid questionnaires were retained, yielding an effective response rate of 96.17%. To reduce evaluation apprehension and socially desirable responding, participation was voluntary and the questionnaire was completed anonymously. Respondents were informed that there were no correct or incorrect answers, that their responses would remain confidential, and that the data would be used solely for academic purposes. All participants received the same instructions, and the questionnaire used clearly worded multi-item measures adapted from previously validated instruments.

**Figure 2:**
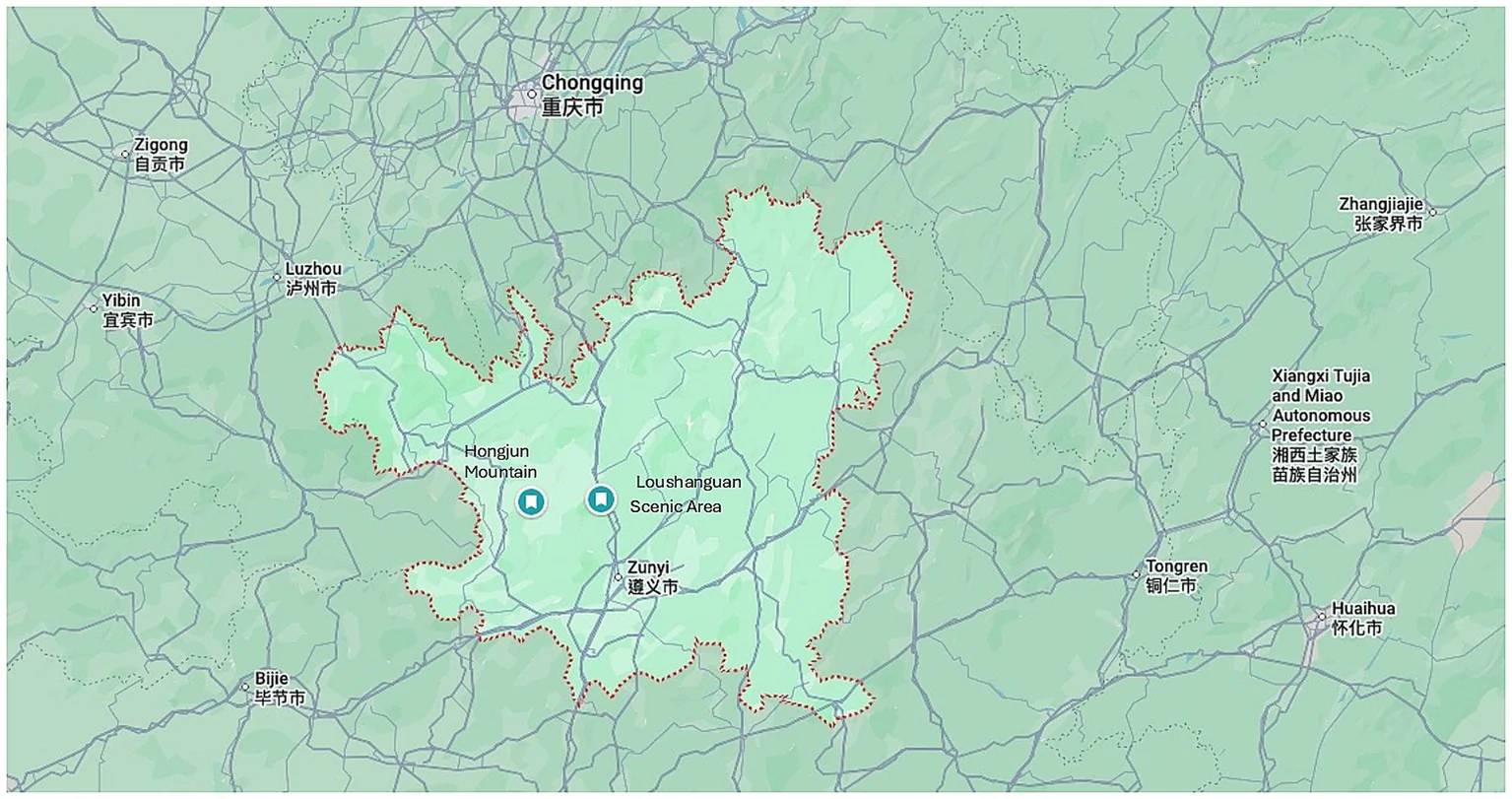
Location of hiking routes at Hongjun Mountain and Loushanguan Scenic Area in Zunyi, Guizhou, China. The image was obtained from Google Maps.

The hiking trail at Loushanguan Scenic Area is approximately 5 kilometers long and passes through forested areas, mountainous terrain, and culturally significant sites such as the Loushanguan Battle Monument and Mao Zedong’s poetry stele. Similarly, the trail at Hongjun Mountain measures about 5 kilometers and passes through Red Army Street and Fenghuangshan Park. With a gentle slope and various scenic viewpoints and rest facilities, it offers both fitness and historical education value. Personnel providing visitor assistance, guidance, interpretation, and related services were present at both study sites. Respondents could therefore observe or encounter these human-supportive elements during their visit, although the degree of direct interaction varied among participants. Similarly, both destinations provided or were connected to activity opportunities such as scenic viewing, photography, nature appreciation, local food experiences, and other outdoor activities, although visitors could choose whether and how extensively to participate in them. These site characteristics supported the use of perception-based measures for hospitality culture and experiential activities. These two sites thus provided rich and authentic field settings for this study. The two hiking destinations, Hongjun Mountain and Loushanguan Scenic Area, are located in Zunyi, Guizhou, China, as shown in Figure 2.

### 3.3 Data analysis

Data were analysed using SPSS 22.0 and AMOS 25.0. Descriptive statistics were first used to summarise the participants’ demographic characteristics and examine the distributions of the study variables. A principal component analysis with Varimax rotation was conducted to explore the dimensional structure of the measurement items. Confirmatory factor analysis was subsequently performed to assess the fit of the seven-factor measurement model, internal consistency, convergent validity, and discriminant validity.

Structural equation modelling was then used to test the hypothesised relationships among the four hiking tourscape dimensions, place dependence, place identity, and psychological restoration. The analysis examined the direct associations of the hiking tourscape dimensions with psychological restoration, place dependence, and place identity, as well as the associations of place dependence and place identity with psychological restoration. Finally, bootstrap analysis with 5,000 resamples was conducted to evaluate the indirect associations of the four hiking tourscape dimensions with psychological restoration through place dependence and place identity. An indirect association was considered statistically significant when its bias-corrected 95% confidence interval did not include zero.

### 3.4 Ethical considerations

This study was conducted in accordance with ethical research standards. The research protocol was reviewed and approved by the Institutional Review Board of the affiliated institution (Approval No. QZSYLL202415). Prior to data collection, all participants were informed of the purpose of the study, the voluntary nature of participation, and their right to withdraw at any time without penalty. Informed consent was obtained from all respondents before participation. The questionnaires were completed anonymously, and the data were used solely for academic research purposes. Participant privacy and confidentiality were protected throughout the research process.

### 3.5 Exploratory factor analysis

Exploratory factor analysis was conducted using principal component analysis with Varimax rotation, retaining factors with eigenvalues greater than 1. The Kaiser–Meyer–Olkin measure indicated that the data were suitable for factor analysis (KMO = 0.920), and Bartlett’s test of sphericity was statistically significant, *χ*^2^(496) = 8948.059, *p* < 0.001.

Seven factors were extracted, accounting for 71.58% of the total variance. The rotated factor loadings ranged from 0.694 to 0.875, with all retained loadings exceeding the recommended threshold of 0.60. The seven factors represented psychological restoration (7 items), place identity (5 items), natural dimension (5 items), experiential activities (5 items), atmosphere dimension (4 items), place dependence (3 items), and hospitality culture (3 items). All items loaded clearly on their respective factors, and no substantial cross-loadings were observed in the rotated component matrix.

Notably, the eight items originally used to assess place attachment were separated into two distinct factors: three items representing place dependence and five items representing place identity. These findings support the multidimensional treatment of place attachment and provide the empirical basis for modelling place dependence and place identity as separate constructs in the subsequent confirmatory factor analysis and structural equation modelling. The results of the exploratory factor analysis are presented in Table 2.

**Table 2:** Exploratory factor analysis results.

Items	PD	PI	PR	ND	EA	AD	HC
PD1: This place is the most suitable for my leisure and relaxation.	0.820						
PD2: Compared to other places, I prefer this place.	0.767						
PD3: Compared to other places, the leisure facilities here better meet my needs.	0.828						
PI1: This place gives me a sense of satisfaction that other places do not.		0.826					
PI2: I strongly identify with this place.		0.820					
PI3: This place is very special to me.		0.843					
PI4: I feel deeply attached to this place.		0.840					
PI5: The unique lifestyle and customs of this place attract me.		0.875					
ND1: The natural scenery of the hiking destination is very charming.				0.790			
ND2: The natural elements of the hiking destination are rich in functions.				0.798			
ND3: The natural landscape and cultural features of the hiking destination are harmoniously integrated.				0.700			
ND4: The hiking destination gives a strong sense of escaping from everyday life.				0.781			
ND5: The scenery of the hiking destination met my expectations.				0.758			
EA1: The hiking destination offers activities for enjoying natural scenery.					0.705		
EA2: The hiking destination allows for camping and outdoor cooking activities.					0.802		
EA3: The hiking destination offers opportunities for photography and creative activities.					0.737		
EA4: The hiking destination provides activities such as hiking and river tracing.					0.712		
EA5: The hiking destination offers opportunities to taste local agricultural products.					0.809		
AD1: The hiking destination has a strong interactive atmosphere.						0.847	
AD2: The atmosphere of the hiking destination is very joyful.						0.832	
AD3: The hiking destination has a strong leisure ambiance.						0.802	
AD4: The pace of life at the hiking destination is very leisurely.						0.835	
HC1: The staff organizing this hiking tour were enthusiastic in their service.							0.806
HC2: The staff organizing this hiking tour were considerate and attentive.							0.788
HC3: The service staff of this hiking tour were friendly and approachable.							0.784
PR1: I felt relieved from stress.			0.855				
PR2: I achieved physical and mental balance.			0.742				
PR3: I felt inner peace.			0.813				
PR4: I felt comfortable and at ease.			0.783				
PR5: It helped me forget my worries.			0.694				
PR6: I felt refreshed and renewed.			0.749				
PR7: I felt full of energy and vitality.			0.826				

PD, Place Dependence; PI, Place Identity; PR, Psychological Restoration; ND, Natural Dimension; EA, Experiential Activities; AD, Atmosphere Dimension; HC, Hospitality Culture.

## 4 Results

### 4.1 Participants’ demographics

Table 3 presents the participants’ demographic characteristics. Of the 452 valid respondents, 66.6% were female and 33.4% were male. Most participants were aged between 18 and 30 years (*n* = 340, 75.2%), followed by those aged 31–45 years (*n* = 64, 14.2%), 46–64 years (*n* = 42, 9.3%), and 65 years or older (*n* = 6, 1.3%). Furthermore, most participants (*n* = 357, 79.0%) held a bachelor’s degree, and 52 participants (11.5%) had completed postgraduate education. Regarding monthly income, 35.2% reported earning RMB 3,000–6,000, while 12.8% earned more than RMB 9,000 per month.

**Table 3:** Participants’ demographics (*n* = 452).

Demographic variable	%
Gender	
Male	33.4%
Female	66.6%
Monthly income	
Below 3,000 RMB	33.8%
3,000–6,000 RMB	35.2%
6,000–9,000 RMB	18.1%
Above 9,000 RMB	12.8%
Age	
18–30 years	75.2%
31–45 years	14.2%
46–64 years	9.3%
65 years or older	1.3%
Education	
Junior high or below	3.1%
High school	6.4%
University	79%
Master’s or above	11.5%

### 4.2 Measure validation

Confirmatory factor analysis was conducted to assess the reliability and validity of the seven-factor measurement model comprising place dependence, place identity, natural dimension, atmosphere dimension, hospitality culture, experiential activities, and psychological restoration. The measurement model demonstrated a very good fit to the data, *χ*^2^(443) = 488.739, *p* = 0.066, *χ*^2^/df = 1.103, GFI = 0.940, AGFI = 0.928, CFI = 0.995, TLI = 0.994, and RMSEA = 0.015. These results indicate that the proposed seven-factor measurement model adequately represented the observed data.

Convergent validity was evaluated using standardised factor loadings, average variance extracted (AVE), and composite reliability (CR). As shown in Table 4, all standardised factor loadings were statistically significant and ranged from 0.658 to 0.905. The AVE values ranged from 0.553 to 0.706, exceeding the recommended threshold of 0.50, while the CR values ranged from 0.847 to 0.927, exceeding the recommended threshold of 0.70. Cronbach’s alpha values ranged from 0.844 to 0.926, indicating good internal consistency across all constructs. These findings support the convergent validity and reliability of the measurement model.

**Table 4:** Standardised factor loadings, reliability, and convergent validity.

Items	Factor loading	Cronbach’s α	AVE	CR
Place dependence		0.844	0.649	0.847
PD1: This place is the most suitable for my leisure and relaxation.	0.844***			
PD2: Compared with other places, I prefer this place.	0.734***			
PD3: Compared with other places, the leisure facilities here better meet my needs.	0.834***			
Place identity		0.891	0.638	0.898
PI1: This place gives me a sense of satisfaction that other places do not.	0.778***			
PI2: I strongly identify with this place.	0.768***			
PI3: This place is very special to me.	0.802***			
PI4: I feel deeply attached to this place.	0.787***			
PI5: The unique lifestyle and customs of this place attract me.	0.857***			
Natural dimension		0.881	0.601	0.882
ND1: The natural scenery of the hiking destination is very charming.	0.823***			
ND2: The natural elements of the hiking destination are rich in functions.	0.843***			
ND3: The natural landscape and cultural features of the hiking destination are harmoniously integrated.	0.660***			
ND4: The hiking destination gives a strong sense of escaping from everyday life.	0.828***			
ND5: The scenery of the hiking destination met my expectations.	0.704***			
Atmosphere dimension		0.905	0.706	0.906
AD1: The hiking destination has a strong interactive atmosphere.	0.864***			
AD2: The atmosphere of the hiking destination is very joyful.	0.834***			
AD3: The hiking destination has a strong leisure ambience.	0.803***			
AD4: The pace of life at the hiking destination is very leisurely.	0.858***			
Hospitality culture		0.871	0.705	0.876
HC1: The staff organising this hiking tour were enthusiastic in their service.	0.893***			
HC2: The staff organising this hiking tour were considerate and attentive.	0.890***			
HC3: The service staff of this hiking tour were friendly and approachable.	0.724***			
Experiential activities		0.857	0.553	0.859
EA1: The hiking destination offers activities for enjoying natural scenery.	0.661***			
EA2: The hiking destination allows for camping and outdoor cooking activities.	0.838***			
EA3: The hiking destination offers opportunities for photography and creative activities.	0.708***			
EA4: The hiking destination provides activities such as hiking and river tracing.	0.658***			
EA5: The hiking destination offers opportunities to taste local agricultural products.	0.831***			
Psychological restoration		0.926	0.645	0.927
PR1: I felt relieved from stress.	0.905***			
PR2: I achieved physical and mental balance.	0.744***			
PR3: I felt inner peace.	0.834***			
PR4: I felt comfortable and at ease.	0.809***			
PR5: It helped me forget my worries.	0.708***			
PR6: I felt refreshed and renewed.	0.771***			
PR7: I felt full of energy and vitality.	0.833***			

***p < 0.001.

Importantly, place dependence and place identity each demonstrated satisfactory reliability and convergent validity when modelled as separate constructs. Place dependence had a Cronbach’s alpha of 0.844, an AVE of 0.649, and a CR of 0.847, whereas place identity had a Cronbach’s alpha of 0.891, an AVE of 0.638, and a CR of 0.898. Together with the exploratory factor analysis, these results support the treatment of place dependence and place identity as empirically distinct dimensions within the broader conceptual domain of place attachment. They were therefore retained as separate constructs in the subsequent structural and mediation analyses.

This modelling decision was also consistent with the theoretical distinction between the two constructs. Place dependence reflects functional suitability and comparative preference, whereas place identity reflects symbolic, emotional, and self-related meanings. Given their distinct factor structure, satisfactory discriminant validity, and low nonsignificant correlation, place dependence and place identity were not combined into a single overall place attachment construct. Treating them separately allowed the subsequent analyses to examine whether the two dimensions had different antecedents, direct associations with psychological restoration, and mediating roles.

Discriminant validity was assessed using the Fornell–Larcker criterion (Fornell and Larcker, 1981). Pearson correlation coefficients were calculated using the mean scores of the seven constructs. As shown in Table 5, the square root of the AVE for each construct exceeded all of its correlations with the other constructs, indicating adequate discriminant validity. In particular, the correlation between place dependence and place identity was low and nonsignificant (*r* = 0.046, *p* = 0.324), further supporting their treatment as distinct constructs.

**Table 5:** Pearson correlations and square roots of AVEs.

Construct	PD	PI	ND	AD	HC	EA	PR
Place Dependence (PD)	**0.806**						
Place Identity (PI)	0.046	**0.799**					
Natural Dimension (ND)	0.418***	0.033	**0.775**				
Atmosphere Dimension (AD)	0.387***	0.065	0.401***	**0.840**			
Hospitality Culture (HC)	0.413***	0.049	0.413***	0.386***	**0.840**		
Experiential Activities (EA)	0.333***	0.062	0.406***	0.349***	0.428***	**0.744**	
Psychological Restoration (PR)	0.409***	0.085	0.450***	0.397***	0.515***	0.412***	**0.803**

Diagonal entries in bold represent the square roots of the average variance extracted. Off-diagonal entries represent Pearson correlation coefficients calculated using the mean scores of the constructs. * p < 0.05, ** p < 0.01, *** *p* < 0.001.

### 4.3 Data normality assessment

Prior to structural equation modelling, the distributions of the composite scores for the seven study constructs were examined using skewness and kurtosis statistics. As shown in Table 6, the skewness values ranged from −0.815 to 0.189, while the kurtosis values ranged from −1.695 to 0.448. These values were within the commonly recommended thresholds of an absolute skewness value below 2 and an absolute kurtosis value below 7 (Kline, 2023), indicating no serious departures from univariate normality.

**Table 6:** Descriptive statistics and normality indicators of study variables (*n* = 452).

Variable	Mean	SD	Skewness	Kurtosis
Place dependence	3.18	1.21	−0.06	−1.55
Place identity	3.60	0.69	−0.82	0.45
Natural dimension	3.04	1.12	−0.03	−1.57
Atmosphere dimension	3.12	1.28	−0.22	−1.59
Hospitality culture	2.92	1.31	0.19	−1.61
Experiential activities	2.92	1.07	0.06	−1.58
Psychological restoration	2.86	1.14	0.12	−1.70

At the individual-item level, skewness values ranged from −0.680 to 0.320 and kurtosis values ranged from −1.605 to 0.608, which also indicated acceptable univariate distributions. These findings supported the use of maximum likelihood estimation in the subsequent confirmatory factor and structural equation analyses. However, acceptable univariate normality does not by itself establish multivariate normality; therefore, the structural results were additionally evaluated using bootstrap confidence intervals.

### 4.4 Common method bias assessment

Because all variables were measured using self-reported questionnaires collected at a single point in time, potential common method bias was assessed using Harman’s single-factor test. All 32 measurement items were entered into an unrotated principal component analysis. The results showed that seven factors had eigenvalues greater than 1, and the first factor explained 31.39% of the total variance, which was below the commonly used threshold of 50%. These findings suggest that common method bias was unlikely to be a serious concern in this study. Nevertheless, the possibility of common method variance cannot be completely ruled out due to the cross-sectional self-report design.

### 4.5 Hypothesis testing

**Figure 3:**
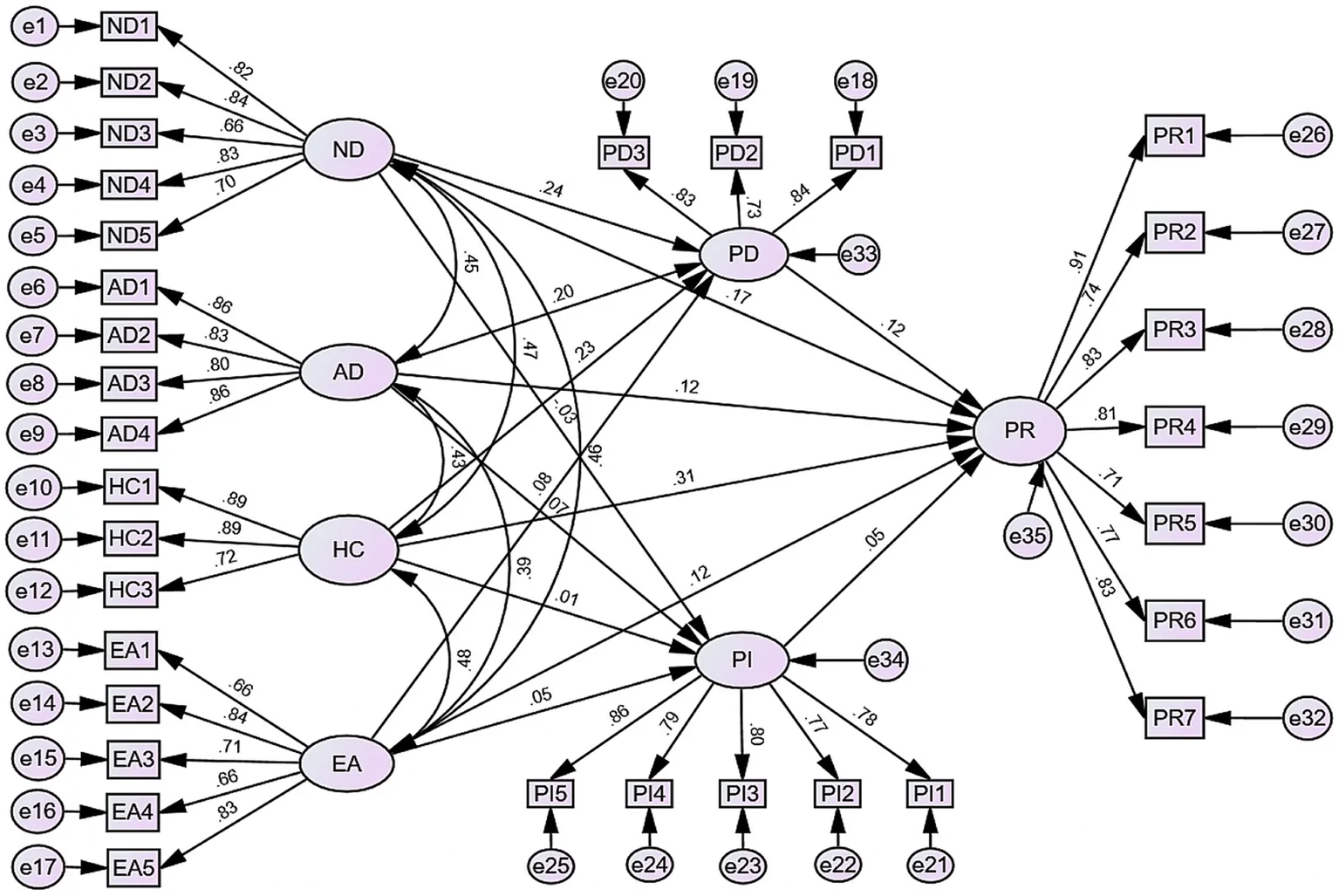
The structural equation model.

Structural equation modelling was conducted to examine the hypothesised relationships among the four hiking tourscape dimensions, place dependence, place identity, and psychological restoration. The structural model demonstrated a good fit to the data, *χ^2^*(444) = 488.748, *p* = 0.070, *χ^2^/df* = 1.101, RMSEA = 0.015, CFI = 0.995, TLI = 0.994, GFI = 0.940, AGFI = 0.928, and NFI = 0.947. The *χ^2^/df* ratio was below 2, the RMSEA was below 0.06, and the CFI and TLI exceeded 0.95, indicating a close fit between the hypothesised model and the observed data. Although the NFI value was slightly below 0.95, it remained close to the recommended criterion and, when considered together with the other fit indices, did not indicate a meaningful model-fit problem. The standardised structural relationships are presented in Figure 3 and Table 7.

**Table 7:** Results of direct hypothesis testing.

Hypothesis	Path	Standardised path coefficient (*β*)	*p*-value	Result
H1a	Natural dimension → psychological restoration	0.174	0.008	Supported
H1b	Atmosphere dimension → psychological restoration	0.117	0.048	Supported
H1c	Hospitality culture → psychological restoration	0.305	< 0.001	Supported
H1d	Experiential activities → psychological restoration	0.123	0.036	Supported
H2a	Place dependence → psychological restoration	0.119	0.061	Not supported
H2b	Place identity → psychological restoration	0.049	0.201	Not supported
H3a	Natural dimension → place dependence	0.237	< 0.001	Supported
H3b	Atmosphere dimension → place dependence	0.202	0.004	Supported
H3c	Hospitality culture → place dependence	0.231	0.001	Supported
H3d	Experiential activities → place dependence	0.083	0.186	Not supported
H4a	Natural dimension → place identity	−0.027	0.671	Not supported
H4b	Atmosphere dimension → place identity	0.066	0.264	Not supported
H4c	Hospitality culture → place identity	0.013	0.867	Not supported
H4d	Experiential activities → place identity	0.050	0.428	Not supported

*p < 0.05, **p < 0.01, ***p < 0.001.

The results showed that all four hiking tourscape dimensions were positively associated with psychological restoration. The natural dimension was positively associated with psychological restoration (*β* = 0.174, *p* = 0.008), as were the atmosphere dimension (*β* = 0.117, *p* = 0.048), hospitality culture (*β* = 0.305, *p* < 0.001), and experiential activities (*β* = 0.123, *p* = 0.036). Thus, H1a, H1c, and H1d were supported, while H1b was supported but should be interpreted as marginal due to its *p*-value being close to 0.05. Among these predictors, hospitality culture showed the strongest direct association with psychological restoration.

Place dependence showed a positive but nonsignificant association with psychological restoration (*β* = 0.119, *p* = 0.061); therefore, H2a was not supported. Similarly, the association between place identity and psychological restoration was not statistically significant (*β* = 0.049, *p* = 0.201), and H2b was not supported. These findings suggest that place dependence may have a limited or marginal role in explaining hikers’ psychological restoration, whereas place identity did not appear to play a meaningful role in the present model.

The natural dimension (*β* = 0.237, *p* < 0.001), atmosphere dimension (*β* = 0.202, *p* = 0.004), and hospitality culture (*β* = 0.231, *p* = 0.001) were positively associated with place dependence. Accordingly, H3a, H3b, and H3c were supported. However, experiential activities were not significantly associated with place dependence (*β* = 0.083, *p* = 0.186), and H3d was therefore not supported. These results suggest that perceptions of natural quality, atmosphere, and interpersonal support contributed to hikers’ perceptions that a destination was functionally suitable and preferable for leisure and restorative activities.

None of the four hiking tourscape dimensions was significantly associated with place identity. Specifically, the associations of the natural dimension (*β* = −0.027, *p* = 0.671), atmosphere dimension (*β* = 0.066, *p* = 0.264), hospitality culture (*β* = 0.013, *p* = 0.867), and experiential activities (*β* = 0.050, *p* = 0.428) with place identity were nonsignificant. Therefore, H4a through H4d were not supported. This pattern suggests that favourable evaluations of the immediate hiking environment may contribute to psychological restoration and functional place dependence without necessarily developing into deeper symbolic identification or incorporation of the destination into the visitor’s self-concept.

### 4.6 Bootstrap test for mediation effects

Bootstrap analysis with 5,000 resamples was conducted to examine the indirect associations of the four hiking tourscape dimensions with psychological restoration through place dependence and place identity. The significance of each indirect effect was primarily evaluated by determining whether its bias-corrected 95% confidence interval included zero. An indirect effect was considered statistically significant when the confidence interval did not contain zero. The results are presented in Table 8.

**Table 8:** Bootstrapped indirect effects of place dependence and place identity.

Hypothesis	Indirect path	Indirect effect *β*	Bias-corrected 95% CI	*p*-value	Result
H5a	Natural dimension → Place dependence → Psychological restoration	0.028	[0.006, 0.054]	0.009	Supported
H5b	Atmosphere dimension → Place dependence → Psychological restoration	0.023	[0.005, 0.048]	0.009	Supported
H5c	Hospitality culture → Place dependence → Psychological restoration	0.027	[0.006, 0.055]	0.009	Supported
H5d	Experiential activities → Place dependence → Psychological restoration	0.011	[−0.001, 0.029]	0.081	Not supported
H6a	Natural dimension → Place identity → Psychological restoration	−0.001	[−0.008, 0.006]	0.880	Not supported
H6b	Atmosphere dimension → Place identity → Psychological restoration	0.002	[−0.003, 0.011]	0.493	Not supported
H6c	Hospitality culture → Place identity → Psychological restoration	0.001	[−0.006, 0.008]	0.834	Not supported
H6d	Experiential activities → Place identity → Psychological restoration	0.002	[−0.004, 0.011]	0.541	Not supported

The indirect association between the natural dimension and psychological restoration through place dependence was statistically significant [*β* = 0.028, 95% CI (0.006, 0.054)], supporting H5a. The indirect association of the atmosphere dimension with psychological restoration through place dependence was also significant [*β* = 0.023, 95% CI (0.005, 0.048)], supporting H5b. Similarly, hospitality culture demonstrated a significant indirect association with psychological restoration through place dependence [*β* = 0.027, 95% CI (0.006, 0.055)], supporting H5c.

In contrast, the indirect association between experiential activities and psychological restoration through place dependence was not statistically significant [*β* = 0.011, 95% CI (−0.001, 0.029)]. Because the confidence interval included zero, H5d was not supported.

None of the four indirect effects through place identity was statistically significant. The confidence intervals for the indirect associations of the natural dimension [*β* = −0.001, 95% CI (−0.008, 0.006)], atmosphere dimension [*β* = 0.002, 95% CI (−0.003, 0.011)], hospitality culture [*β* = 0.001, 95% CI (−0.006, 0.008)], and experiential activities [*β* = 0.002, 95% CI (−0.004, 0.011)] with psychological restoration all included zero. Therefore, H6a, H6b, H6c, and H6d were not supported.

## 5 Discussion

This study examined how four dimensions of hiking tourscape were associated with psychological restoration and whether place dependence and place identity provided distinct explanatory pathways. The findings showed that all four hiking tourscape dimensions were directly associated with psychological restoration, although their effect sizes differed. Hospitality culture had the strongest direct association with psychological restoration, followed by natural dimension, experiential activities, and atmosphere dimension. More importantly, the mediation results revealed a selective pattern rather than a uniform place attachment mechanism. Natural dimension, atmosphere dimension, and hospitality culture showed significant specific indirect associations with psychological restoration through place dependence, whereas experiential activities did not. None of the four tourscape dimensions showed significant indirect associations through place identity. These findings suggest that psychological restoration in hiking tourism is shaped mainly by immediate tourscape experiences, while place dependence provides a selective functional pathway for environmental, atmospheric, and interpersonal features. Place identity, by contrast, did not operate as a meaningful explanatory pathway in the present model.

The positive direct associations of all four tourscape dimensions with psychological restoration indicate that restoration in hiking settings extends beyond exposure to nature alone. Natural scenery, vegetation, terrain, and changing landscapes may facilitate fascination, psychological distance from daily demands, and stress reduction, as proposed by Attention Restoration Theory and Stress Recovery Theory (Kaplan and Kaplan, 1989; Kaplan, 1995; Ulrich et al., 1991). This finding is consistent with previous research linking hiking and nature-based recreation with attentional recovery, reduced anxiety, and psychological well-being (Mitten et al., 2018; Nordbø and Prebensen, 2015). However, the results also indicate that natural qualities are only one part of the restorative experience. Atmosphere, hospitality culture, and experiential activities each contributed directly to psychological restoration, suggesting that hiking restoration is produced through a combination of environmental, affective, interpersonal, and participatory conditions.

Hospitality culture showed the strongest direct association with psychological restoration. This finding is notable because restorative environment research has traditionally focused more on natural and physical features than on interpersonal support. Friendly, attentive, and approachable staff may reduce uncertainty regarding routes, facilities, safety, and physical demands, while also increasing reassurance and psychological security. In unfamiliar hiking environments, such support may help visitors feel more comfortable and mentally relaxed. Tourism studies have similarly linked supportive service encounters with emotional well-being, positive affect, and favourable destination experiences (Abdulaziz et al., 2023; Gao and Tong, 2010; Kim and Perks, 2021; Le et al., 2021). This result does not imply that hospitality is universally more restorative than nature, but it suggests that visible and supportive human interaction can be an important complement to environmental qualities in hiking tourism.

Atmosphere and experiential activities also contributed directly to psychological restoration. A joyful and leisurely atmosphere may reduce tension and strengthen the sense of being away, while opportunities for photography, nature observation, outdoor cooking, and other activities may provide novelty, absorption, and temporary disengagement from routine concerns. These findings accord with evidence that positive environmental atmospheres and participatory experiences support emotional comfort and cognitive recovery (Korpela and Hartig, 1996; Korpela et al., 2017; Cui et al., 2025; Song et al., 2025). However, experiential activities were not significantly associated with place dependence and did not produce a significant indirect effect through place dependence. This suggests that activity opportunities may enrich the immediate visit without necessarily making the destination functionally irreplaceable. Photography, outdoor cooking, nature observation, and local food experiences may be enjoyable but may also be available at other hiking sites. Their restorative value may therefore arise mainly from immediate engagement and novelty rather than from comparative destination preference. Because experiential activities were measured as perceived opportunities rather than actual participation, future research should distinguish between availability, awareness, participation, and quality of involvement.

The selective mediation pattern through place dependence deserves particular attention. Natural dimension, atmosphere dimension, and hospitality culture were indirectly associated with psychological restoration through place dependence, suggesting that these features may strengthen restoration not only through immediate experience but also by making the destination appear functionally suitable and preferable for leisure and recovery. Natural qualities may support place dependence because scenery, terrain, vegetation, and ecological features are relatively stable and destination-specific resources. These qualities can make a hiking destination feel suitable for exercise, nature engagement, relaxation, and escape. Similarly, a pleasant atmosphere may enhance the perceived compatibility of the destination with visitors’ recovery goals, while hospitality culture may reduce uncertainty, improve usability, and increase visitors’ confidence in using the destination. In this sense, place dependence reflects a functional mechanism through which environmental quality, situational comfort, and interpersonal support become linked to psychological restoration.

At the same time, place dependence did not show a statistically significant direct association with psychological restoration under the bias-corrected bootstrap criterion. This finding suggests that functional suitability should not be interpreted as a strong independent predictor of restoration when the direct effects of tourscape dimensions are simultaneously considered. Rather, place dependence appears to operate as a limited and selective functional pathway. It may help explain why certain relatively stable or supportive destination features, such as natural quality, atmosphere, and hospitality culture, become connected to restoration, but it does not replace the more immediate sensory, affective, interpersonal, and participatory routes through which hiking experiences support recovery.

Place identity, by contrast, played a very limited role in the present model. None of the four hiking tourscape dimensions significantly predicted place identity, and place identity did not significantly predict psychological restoration. Although personally meaningful places may support restoration (Ratcliffe and Korpela, 2016; Subiza-Pérez et al., 2021), identity-based bonds generally involve self-definition, autobiographical memory, belonging, and long-term emotional continuity (Proshansky et al., 1983; Williams and Vaske, 2003). These qualities may require repeated visitation or enduring personal experience. Because the present study measured current evaluations during an on-site visit and did not include visitation history, residency, or autobiographical meaning in the structural model, the findings may have captured functional preference more effectively than deeper symbolic identification.

The absence of significant paths to place identity is theoretically meaningful. Favourable scenery, atmosphere, hospitality, and activity opportunities may improve enjoyment, comfort, and functional suitability without becoming part of the visitor’s self-concept. Place identity generally develops through accumulated memories, personal history, social relationships, and repeated interpretation of place experiences (Proshansky et al., 1983; Scannell and Gifford, 2010). The absence of a natural dimension to place identity relationship is especially noteworthy because natural settings are often assumed to acquire symbolic meaning (Raymond et al., 2010; Sthapit et al., 2023). In this sample, respondents may have valued the landscape primarily for what it enabled them to do rather than for its role in defining who they were.

This interpretation is reinforced by the low and nonsignificant correlation between place dependence and place identity. Although both constructs are commonly treated as dimensions of place attachment (Williams et al., 1992; Williams and Vaske, 2003), respondents appeared to distinguish functional preference from symbolic identification. They could perceive the destinations as useful and suitable for hiking without viewing them as personally defining. The satisfactory reliability and discriminant validity results also support their treatment as distinct constructs. At the same time, the weak relationship between place dependence and place identity should be examined further in other hiking contexts, especially among repeat visitors, residents, and highly involved outdoor recreation participants.

Overall, the findings refine the role of place attachment in hiking tourism. Hiking tourscape was associated with restoration mainly through direct environmental, affective, interpersonal, and participatory processes. Selected tourscape dimensions also operated through a limited functional pathway via place dependence, whereas place identity did not perform the same role. The results therefore support treating functional suitability and symbolic identification as distinct mechanisms with different antecedents and different relevance to psychological restoration.

### 5.1 Theoretical contributions

This study makes three main theoretical contributions. First, it develops hiking tourscape as a multidimensional framework comprising natural dimension, atmosphere dimension, hospitality culture, and experiential activities. Previous restorative environment research has often focused on natural exposure, landscape perception, or perceived restorativeness as broad environmental evaluations. By examining four distinct tourscape dimensions, this study shows that psychological restoration in hiking settings is associated with a wider set of route-based environmental and experiential conditions. The significant direct associations of all four dimensions with psychological restoration support a view of hiking as an embodied and relational experience in which scenery, ambience, human support, and participatory opportunities jointly shape restorative experience.

Second, the findings clarify how Attention Restoration Theory and Stress Recovery Theory can be integrated with place attachment theory. ART and SRT explain how environmental and experiential cues may be associated with relatively immediate attentional and emotional recovery. In the present study, all four tourscape dimensions were directly associated with psychological restoration, suggesting that restorative responses may arise during the hiking encounter itself through sensory, affective, interpersonal, and participatory processes. Place attachment theory adds a relational explanation, but the results show that this pathway is selective rather than uniform. Natural dimension, atmosphere dimension, and hospitality culture were indirectly associated with psychological restoration through place dependence, whereas experiential activities were not. Because place dependence had only a marginal and nonsignificant direct association with psychological restoration, its role should be understood as a limited and complementary functional pathway rather than as the central mechanism of restoration.

Third, the study demonstrates the importance of separating place dependence from place identity. Treating place attachment as a single construct would have obscured the finding that functional suitability and symbolic identification played different roles. Place dependence was associated with several tourscape dimensions and provided significant specific indirect pathways for natural dimension, atmosphere dimension, and hospitality culture. In contrast, place identity neither predicted psychological restoration nor mediated any of the proposed relationships. These findings suggest that the functional and symbolic dimensions of place attachment can have different antecedents, consequences, and explanatory relevance. This distinction offers a more precise theoretical account of person-place relationships in tourism and outdoor recreation.

### 5.2 Practical implications

The findings have practical implications for hiking destination planning and management. Because all four tourscape dimensions were directly associated with psychological restoration, managers should avoid focusing exclusively on natural scenery. Protecting vegetation, ecological diversity, landscape continuity, and scenic viewpoints remains essential, but restorative hiking experiences also depend on atmosphere, interpersonal support, and opportunities for meaningful participation. At the same time, the significant indirect effects of natural dimension, atmosphere dimension, and hospitality culture through place dependence suggest that some destination features may support restoration not only by producing immediate comfort and recovery, but also by strengthening visitors’ perceptions that the destination is suitable, usable, and preferable for leisure and restoration. Management strategies should therefore distinguish between features that enhance immediate restorative experience and features that also strengthen functional place dependence.

Natural resources should be managed as both restorative assets and functional resources. Scenic landscapes, vegetation, terrain, ecological diversity, and quiet viewing areas can help visitors experience fascination, escape, and relaxation, while also reinforcing the perception that the destination is suitable for exercise, nature engagement, and psychological recovery. Managers can strengthen this functional connection by designing scenic interpretation points, ecological explanation boards, shaded rest areas, quiet viewing spaces, and route narratives that help visitors understand and experience the restorative value of the landscape. These strategies may increase the compatibility between the natural setting and visitors’ recovery goals.

Atmosphere and hospitality culture should also be managed as part of the destination’s restorative and functional environment. A calm, joyful, and leisurely atmosphere can reduce tension and support immediate relaxation, while good route organisation can make the destination easier and more comfortable to use. Managers should therefore control crowding, reduce unnecessary noise, maintain cleanliness, provide comfortable rest spaces, and arrange facilities at an appropriate pace along the route. Clear signage, smooth circulation, and well-designed rest points can help visitors move through the trail with less uncertainty and greater psychological comfort. Similarly, hospitality culture should be strengthened through visible and supportive human assistance. Approachable staff, clear route information, safety guidance, assistance points, and accessible interpretive services can reduce uncertainty, increase visitors’ confidence, and strengthen the perception that the site is functionally suitable for leisure, exercise, and restoration. Staff training should include not only operational knowledge but also attentive communication and sensitivity to visitors with different levels of hiking experience.

Experiential activities should be managed differently. Although experiential activities were directly associated with psychological restoration, they were not significantly associated with place dependence and did not produce a significant indirect effect through place dependence. This suggests that activities such as photography points, nature observation, cultural interpretation, local food experiences, and appropriately managed outdoor activities may be most useful as immediate restorative enhancers. They can increase novelty, absorption, enjoyment, and attentional diversion during the visit, but managers should not assume that simply increasing the number of activities will automatically create functional destination attachment. To increase their value, such activities should be distinctive, well integrated with the trail, and clearly connected to the destination’s specific natural and cultural resources.

The nonsignificant place identity pathway also provides useful guidance for long-term destination management. Because no tourscape dimension was significantly associated with place identity, short-term improvements in scenery presentation, service quality, or activity provision may not be sufficient to generate symbolic identification. Managers seeking to cultivate deeper person-place bonds should consider longer-term strategies, such as repeated-visit programmes, seasonal trail events, storytelling, cultural interpretation, community participation, commemorative experiences, and opportunities for visitors to build personal memories with the destination over time. These strategies should be evaluated longitudinally rather than assumed to result directly from a favourable single visit.

Overall, the practical implications should be understood in terms of differentiated mechanisms. Natural quality, atmosphere, and hospitality culture can be managed both as immediate restorative resources and as contributors to functional place dependence. Experiential activities appear more useful for enhancing immediate comfort, enjoyment, engagement, and relaxation during the visit. Place identity may require longer-term and more personally meaningful engagement. This distinction allows hiking destination managers to design more evidence-based strategies for supporting psychological restoration.

### 5.3 Limitations and future research

Several limitations should be considered when interpreting the findings. First, the cross-sectional design does not establish causal or temporal relationships. Although the model was theoretically specified from hiking tourscape to place dependence, place identity, and psychological restoration, individuals who feel more restored may also evaluate the destination more positively. Longitudinal studies, repeated measurement designs, and field experiments are needed to examine how restorative responses and person-place relationships develop over time.

Second, convenience sampling was used at two destinations in Zunyi, China, and the sample was predominantly young and university educated. The findings may not generalise to older hikers, highly experienced hikers, residents, international visitors, or users of destinations with different environmental and cultural characteristics. Future research should use more diverse and probability-based samples and compare urban trails, rural routes, protected areas, and culturally significant hiking destinations.

Third, place identity may require repeated visits and accumulated personal meaning, but visitation history, length of relationship with the destination, local residency, and hiking involvement were not incorporated into the structural model. These factors may help explain why place identity was not associated with the tourscape dimensions or psychological restoration. Future studies should examine whether repeat visitation, autobiographical memory, social bonding, and activity commitment moderate or precede the development of place identity.

Fourth, the study relied on self-reported perceptions collected at a single point in time. Although procedural steps were taken to reduce evaluation apprehension and common method bias, and Harman’s single-factor test suggested that common method bias was unlikely to be a serious concern, common method variance cannot be completely ruled out. Recall, current mood, physical fatigue, weather, and crowding may also have influenced responses. Future research could combine surveys with physiological measures, behavioural observation, environmental indicators, experience sampling, or pre- and post-hike assessments to provide a more comprehensive evaluation of restoration.

Fifth, experiential activities were measured as perceived opportunities available at the destination rather than verified participation in each activity. This approach was appropriate for assessing the perceived experiential environment, but it does not distinguish between availability, awareness, actual participation, and quality of involvement. Future research could examine which activities visitors undertake, how deeply they participate, and whether participation intensity affects restoration, place dependence, and place identity.

Finally, the findings are based on the proposed seven-factor measurement and structural model. Although place dependence and place identity demonstrated satisfactory reliability and discriminant validity, the unusually weak relationship between them merits further examination. Future studies should replicate the measurement structure in other hiking contexts, test measurement invariance across visitor groups, and investigate whether alternative constructs such as social bonding, familiarity, place memory, nature connectedness, or destination involvement provide additional explanations of restorative hiking experience.

## 6 Conclusion

This study examined how four dimensions of hiking tourscape were associated with psychological restoration and whether place dependence and place identity provided distinct explanatory pathways in hiking destinations in China. The findings showed that natural dimension, atmosphere dimension, hospitality culture, and experiential activities were all directly and positively associated with psychological restoration, indicating that restorative hiking experiences arise from a broader combination of environmental, affective, interpersonal, and activity-related features rather than from natural exposure alone. Among these dimensions, hospitality culture showed the strongest direct association with psychological restoration, highlighting the importance of supportive human interaction in restorative hiking experiences. The results further indicated that place dependence and place identity played different roles. Natural dimension, atmosphere dimension, and hospitality culture were positively associated with place dependence, suggesting that these qualities may strengthen visitors’ perceptions that a destination is functionally suitable and preferable for leisure and recovery; however, place dependence showed only a marginal and nonsignificant direct association with psychological restoration, so its role should be interpreted as limited rather than as a strong direct predictor. Experiential activities were directly associated with psychological restoration but were not significantly associated with place dependence, suggesting that their contribution may arise mainly from immediate engagement, novelty, and temporary detachment during the visit. In contrast, place identity was not significantly associated with psychological restoration, none of the four hiking tourscape dimensions significantly predicted place identity, and no indirect effects through place identity were identified. This pattern suggests that favourable evaluations of a hiking environment may support immediate recovery and functional preference without necessarily producing deeper symbolic identification, which may require repeated visitation, accumulated memories, social connections, or longer-term personal involvement. By distinguishing place dependence from place identity, this study demonstrates that place attachment should not be treated as a uniform mechanism and extends restorative environment research by conceptualising hiking as a route-based and multidimensional experience involving direct environmental, affective, interpersonal, and participatory pathways to restoration, as well as a more limited functional pathway through place dependence. The findings should nevertheless be interpreted cautiously because the study used cross-sectional data, convenience sampling, and a predominantly young and university-educated sample from two destinations. Future research should adopt longitudinal or repeated-visit designs, include more diverse destinations and visitor groups, and examine visitation history, autobiographical meaning, social bonding, and actual activity participation to clarify how place dependence and place identity develop and contribute to psychological restoration over time.

## Data Availability

The original contributions presented in the study are included in the article/supplementary material, further inquiries can be directed to the corresponding author.
